# Mixed methods implementation research to understand success of intensive combination approach to roll back the epidemic in Nigerian adolescents) (iCARE Nigeria) HIV testing uptake and linkage to care among young men focusing on young men who have sex with men in Ibadan

**DOI:** 10.1186/s12981-023-00574-4

**Published:** 2023-10-30

**Authors:** Lisa R. Hirschhorn, Adedotun Adetunji, Aima A. Ahonkhai, Bibilola Oladeji, Olutosin A. Awolude, Lisa M. Kuhns, Jude Onumabor, Kehinde M. Kuti, Olayinka Omigbodun, Amy K. Johnson, Ogochukwu Okonkwor, Babafemi Taiwo, Robert Garofalo

**Affiliations:** 1 Department of Medical Social Sciences, Feinberg School of Medicine, Northwestern University, 625 N Michigan Ave, 14-013, Chicago, IL 60611 USA; 2 Feinberg School of Medicine, Ryan Family Center for Global Primary Care, Robert J Havey Institute of Global Health, Northwestern University, Chicago, USA; 3 Department of Family Medicine, University College Hospital, Ibadan, Nigeria; 4 Department of Medicine, Infectious Diseases, Vanderbilt University Medical Center, Nashville, TN USA; 5 Vanderbilt Institute for Global Health, Vanderbilt University Medical Center, Nashville, TN USA; 6 Department of Psychiatry, College of Medicine, University of Ibadan, Ibadan, Nigeria; 7 Department of Obstetrics and Gynecology, College of Medicine, University of Ibadan, Ibadan, Nigeria; 8 Infectious Disease Institute, College of Medicine, University of Ibadan, Ibadan, Nigeria; 9 Department of Pediatrics, Northwestern University Feinberg School of Medicine, Chicago, IL USA; 10 Division of Adolescent and Young Adult Medicine, Ann & Robert H. Lurie Children’s Hospital of Chicago, Chicago, IL USA; 11 Initiative for the Advancement of Improved Health and Development, I-AIHD, Ibadan, Nigeria; 12 Staff Medical Services Department, University College Hospital, Ibadan, Nigeria; 13 Department of Preventive Medicine, Northwestern University Feinberg School of Medicine, Chicago, IL USA; 14 Division of Infectious Diseases and Center for Global Health, Northwestern University, Chicago, IL USA; 15 Department of Psychiatry, College of Medicine, University of Ibadan, Ibadan, Nigeria; 16 Centre for Child and Adolescent Mental Health, College of Medicine,, Ibadan University, Ibadan, Nigeria

**Keywords:** HIV, Nigeria, Implementation science, Men who have sex with men, HIV testing

## Abstract

**Background:**

HIV seroprevalence in Nigeria is increasing among men who have sex with men (MSM) from 14% to 2007 to 23% in 2014, threatening progress towards ending the epidemic in the country. Expanding access to HIV testing and linkage to care for key populations, like young MSM (YMSM), is critical to end the HIV epidemic in Nigeria. The Intensive Combination Approach to Roll Back the Epidemic in Nigerian Adolescents (iCARE Nigeria) pilot intervention successfully implemented a combination of evidence-based interventions utilizing peer navigators and popular social media apps and platforms to reach young men at risk for HIV exposure, including YMSM. We conducted sequential mixed methods explanatory implementation research to expand on the previously reported effectiveness and implementation outcomes and to explore the determinants and strategies which contributed to primary study results.

**Methods:**

We conducted key informant interviews and focus group discussions with 2 peer navigators and 3 study staff at the end of the pilot. We used directed content analysis to understand the quantitative results from the pilot. Using the Implementation Research Logic Model, we were able to identify and map strategies through mechanisms of action from barriers addressed to the reported implementation outcomes including feasibility, acceptability fidelity and adoption.

**Results:**

We found that iCARE Nigeria’s pilot intervention implementers reported high feasibility, acceptability fidelity and adoption were associated with implementation of strategies which addressed many challenging contextual factors, including social stigma, online social networking, legal barriers surrounding MSM behavior, and the COVID-19 pandemic. These strategies included integration of stakeholders’ interests, selection of experienced peer navigators including from the targeted population, training and supportive supervision using an implementation guide, ensuring safety (COVID and legal) and identification of clinics serving the targeted population.

**Conclusion:**

Mixed methods using implementation research frameworks provided insights into the strategies and barriers and facilitators they addressed which may explain the success of the pilot. These results can inform strategies needed to scale-up the intervention to youth including YMSM in other areas in Nigeria and the region.

*Trial registration* ISRCTN: ISRCTN94590823, 10.1186/ISRCTN94590823

**Supplementary Information:**

The online version contains supplementary material available at 10.1186/s12981-023-00574-4.

## Introduction

Nigeria has had success in decreasing HIV seroprevalence by one half (from 2.8 to 1.4%) among adults aged 15–49 years. However, HIV seroprevalence actually increased among young men who have sex with men (YMSM) from 14% to 2007 to 23% in 2014, threatening progress towards ending the epidemic in the country [1–3]. While the HIV treatment program has continued to increase coverage throughout Nigeria, expanding access to HIV testing and linkage to and retention in appropriate care for young men, including YMSM is critical to end the HIV epidemic in Nigeria [3].

The Intensive Combination Approach to Rollback the Epidemic in Nigerian Adolescents (iCARE Nigeria) testing pilot intervention in Ibadan successfully implemented a combination of evidence-based interventions utilizing peer navigators and popular social networking apps and platforms to reach young men at risk for HIV exposure, including YMSM [4]. These evidence-based interventions were adapted by the iCARE Nigeria team to reflect local context in Nigeria [5]. Over a 12-month pilot study, the combination intervention resulted in testing of 339 young men, with most (83.5%) referred through social media platforms and almost all tested in community or home settings. High rates of reaching the targeted population were seen with 10.6% testing HIV-positive, a threefold increase over historical trends. The majority (86.1% n = 31) of HIV-positive YMSM were linked to care. The study also reported high rates of fidelity, acceptability, and satisfaction [4].

Implementation science is defined as “the scientific study of methods to promote the systematic uptake of research findings and other evidence-based practice into routine practice and, hence, to improve the quality and effectiveness of health services” [6]. Implementation research uses methods including mixed methods to understand the barriers and facilitators (called determinants) and strategies used to address these determinants which influence the implementation outcomes and effectiveness of interventions which have been shown to be efficacious in study settings (called evidence-based interventions) [7]. The Implementation Research Logic Model, combines a number of implementation science frameworks and is designed to guide understanding of the results of implementation through mapping strategies to the barriers and facilitators they target and the mechanisms through which the outcomes are potentially influenced [8]. Such understanding of the relationships between barriers and facilitators, strategies and outcomes will be important to inform future work to adapt for other settings and scale-up iCARE Nigeria in the country and in the region.

Our objective, therefore, was to use implementation science mixed methods to identify and map the key determinants (facilitators and barriers), and implementation strategies that were associated with the favorable implementation and effectiveness outcomes for the iCARE intervention reported by Garofalo et al. [4]. We then used the Implementation Research Logic Model to provide a map through which the results can be understood to inform future work to adapt for other settings and scale-up iCARE Nigeria in the country and in the region.

## Methods

### Study setting

The iCARE Nigeria testing intervention was implemented within the HIV care programs of the Infectious Disease Institute of the College of Medicine, University of Ibadan, and the University College Hospital, both in Ibadan, the third-largest city in Nigeria by population.

### Intervention and population

The intervention is described in detail elsewhere [4]. Briefly, the iCARE Nigeria testing intervention combined social media outreach including networking apps and peer navigation to reach young men (aged 15–24 years) to facilitate both HIV testing and, for those testing positive, linkage to HIV care. The design included a public health approach, to assess the intervention’s ability to increase HIV testing and diagnosis in young men, focusing on YMSM. Peer navigators were chosen largely from the target population and received training for HIV rapid testing based on Nigerian national guidelines and best practices including an emphasis on safety (for peer navigators and participants), confidentiality, and human rights protections. Additional training focused on the social media component, including setting up social media-based groups for interaction, developing outreach messages, and managing interactions with participants to facilitate relationships and foster a safe “community” for HIV testing. iCARE Nigeria testing was implemented at the same time as iCARE Nigeria treatment, an intervention also using peer-navigators and text messaging to improve viral suppression among youth living with HIV [3].

iCARE Nigeria used a combination of non-MSM specific social media and networks apps (WhatsApp and Facebook) as well as MSM-specific social media platforms that use geolocator technology (Grindr). WhatsApp was used to create a closed social media group which provided a platform for sharing education and discussion on both HIV and non-HIV health topics of interest as identified by the study team and participants, and topics of relevance to HIV testing, prevention, and care. Other strategies at the start of iCARE included stakeholder engagement during the design phase including community-based organizations working with the MSM community, identifying key population-friendly care sites for linkage to care, and supportive supervision of the peer navigators.

### Mixed methods

We conducted a sequential explanatory mixed methods study to expand on the reported effectiveness and implementation outcomes by Garofalo et al. in 2022. We explored how the determinants (barriers and facilitators) and the implementation strategies contributed to primary study implementation outcomes including feasibility, acceptability, adoption, and reach [9]. Quantitative data were used from the previously published article [4] and additional data obtained from surveys of individuals who accepted HIV testing. These included questions about the site where testing was done, process and satisfaction of testing, willingness to refer others to iCARE Nigeria, and any issues with disclosure. Additional quantitative data included client demographics, previous testing, test results and linkage to care. Individual data about sexual behavior, sexual identity or orientation were not collected to ensure safety of participants.

Qualitative data included key informant interviews with a convenience sample of two of the four peer navigators and a focus group discussion with program staff (program manager, and outreach coordinator). Peer navigators were chosen based on willingness to participate and availability for in-person interviews during the COVID-19 pandemic and all consented. The number was limited based on time and COVID-related restrictions. None of the young men who received HIV testing services were included in interviews because this study was conducted post hoc and iCARE Nigeria data were collected in deidentified format, so participants could not be contacted for participation. Interview guides were designed to focus on key potential barriers and facilitators from the Consolidated Framework for Implementation Research [10] domains including inner setting, actors (testing peer navigators), outer setting (community and key population) and the intervention itself, as well as the selected implementation outcomes including adoption, feasibility, fidelity and acceptability (see Additional file 1) [11]. Interviews and focus groups were audio recorded and transcribed for analysis.

The University of Ibadan (19/0123), Ann & Robert H. Lurie Children’s Hospital (protocol 2019-2466) and Northwestern University (STU00207490) approved this study with a waiver of documentation of written consent for both the interviews and abstraction of programmatic data. All individuals gave verbal informed consent priori to interviews.

### Analysis

Descriptive statistics were used to summarize available quantitative data describing implementation outcomes not reported in the Garofalo paper [4] including those in domains of adoption, implementation feasibility and fidelity, and acceptability (see Additional file 1 for definitions) .

We used directed content analysis for qualitative data based on the goals of identifying barriers and facilitators in CFIR domains and strategies associated with predefined implementation outcomes [12]. Transcripts were read and memos created guided by the CFIR domains and selected Proctor’s implementation outcomes. Results from the qualitative interviews were reviewed by two authors (LRH, AA) to discuss emerging explanatory themes within the predefined outcomes and gain agreement. Qualitative results were triangulated with quantitative data to identify areas of confirmation, differences, and explanatory insights. Results were then synthesized to identify barriers and facilitators encountered by the study team and peer navigators, strategies implemented to address them and where adaptations during the study were needed. The data on barriers, facilitators, strategies, and outcomes were linked together through discussion with the team to identify proposed mechanisms to populate the Implementation Research Logic Model. Results were presented to the implementing team for further clarification and input and to facilitate integration into ongoing scale-up activities.

## Results

We interviewed two of the four peer navigators (both male) and the project manager and outreach coordinator. Only participants and interviewers were present, and interviews were done in the project office. No one refused participation. All staff interviewed had been employed on the project for over 1 year. Saturation of themes was found based on a review of interview transcripts. We used the Consolidated criteria for reporting qualitative research (COREQ checklist as recommended for qualitative studies (see Additional file 2) and recommended practices for reporting mixed methods studies [13].

### Implementation outcomes (Table 1)

**Table 1:** Quantitative implementation outcomes for clients and illustrative explanatory qualitative quotes (see Additional file 1 for outcome definitions)

Outcomes	Quantitative results	Illustrative quotes
Feasibility	Testing peer navigators hired and retained, able to test in the community (39% in home, 60% in community) Social media outreach conducted as planned and closed group on generic social media platform initiated and continued weekly, led by peer navigators and program leads	*"And for … testing peer navigator, we make use of that (social media) as a strategy to get to know new people, new members” (peer navigator)* *“after adding them on WhatsApp, you still need to create a mutual rapport with them. Let them know that there is a place you can still add them, whereby they will be receiving daily topic concerning … health (peer navigator)*
Fidelity	100% had pre-test counseling before undergoing rapid testing, followed by post-test counseling based on test results People testing negative for HIV infection: 99.7% (302/303) reminded for follow-up People testing positive for HIV infection :100% (36/36) received confirmatory test and referral for care within 30 days	*"so, I seek their consent first before anything and I move into counselling and after giving their consent for the testing, I counsel with them. I move on to test them and for post-counselling” (peer navigator)*
Adoption	100% of peer navigators participated in social media, conducted in-person outreach, testing, and navigation	*"that I try to familiarize myself with the client., I talk to them about their daily activities if need be and …., so, I seek their consent first before anything and I move into counselling and after giving their consent for the testing, I counsel with them. I move on to test them and for post-counselling … And then, if it’s a negative result, I talk to them about all the preventive and protective measures and … if it’s reactive, I continue with my post-counselling and eventually navigate them for confirmation at any available clinic” (peer navigator)*
Acceptability^a^	99% (336) who agreed to meet with the peer navigator accepted testing by them^a^ Most (83.5%) were referred through iCARE social media 410 young men participated in the WhatsApp group	*"because our peer navigators, they have been trained before we embark on a field work. So, the persuasive power of the peer navigator, and the ability to engage these people, and they see them as being part of them. The participants see the peer navigator as being part of them” (Program Staff)*
Reach	All individual enrolled identified as male at birth and were in the targeted age group	*"of course the social media has really been a successful … strategy and that has really helped our work and all that. Like WhatsApp, Facebook and Grindr.” (peer navigator)* *I do make to them is that “ok, fine I am MSM. We have lots of MSM there in the clinic …. So, and this is what we do, and don’t even look at any other person, look at me as the same community member.”(peer navigator)*
Maintenance	All peer navigators remained in iCARE and providing testing throughout the study	*"So, at the end of the week, we come to review what we have done in the field and even within the uhm in the office. So, planning meetings, review meetings and with team work, those are 2 major factor.* *….there are times we start seeing that they (PNs) become like nonchalant, they are getting tired. They are like err, they don’t want to do, so we just try to put them on their toes. We give them more, like more work to do. [laughter] yes, we do that….And encourage them too” (Program staff)*

^a^Acceptability also included results reported by Garofalo et al. including 93.7% of participants in testing events said they trusted the results of the test to a great extent, with 292 (80.4%) being very satisfied. A total of 292 participants (80.4%) were very satisfied and 68 (18.7%) with test testing process. Almost all (98.6%) who underwent testing would recommend iCARE to a friend. Reach included the HIV test positivity rates of the individuals who agreed to testing at 10.6%, almost fivefold higher than the rates in the general population [4]

Reach was high among individuals who agreed to testing identified as male at birth and in the targeted age group. The social media intervention was also feasible, with ongoing outreach and education through the publicly available apps which included both general (WhatsApp, Facebook) and MSM-specific platforms (Grindr) that supported community building completed as planned. Between June 2019 and April 2020, 410 young men joined the iCARE WhatsApp chat group. Topics included sexually transmitted infections, mental health, condoms, introduction to PrEP and coping with sexually intimate issues among YMSM. Most [83% (283)] of those tested were identified through the social media networking activities.

Feasibility was also established for program implementation and the testing component, with peer navigators hired as planned and retained, with ability to test in the community or home of study participants (Table 1). Fidelity to the protocol for counseling, rapid testing, confirmatory testing, and linkage to care were also high for all steps along the cascade for testing and referral to care if testing positive for HIV. Further confirmation of fidelity was reported during the interviews from the peer navigators on process of pretest counseling, testing, and linkage to care. Adoption by peer navigators was also high with 100% participating in social media outreach, community or home-based testing and linkage of clients who tested positive for HIV into care. Acceptability was also high for meeting and getting tested by the peer navigators. Navigating young men to meet in person for HIV testing took time and was the largest hurdle. Once the men agreed to meet, high acceptability of testing was seen with (99%, 336) of men who met the peer navigators in person accepting testing in the community or in their homes. Maintenance was also see with all PNs remained in the program and providing testing through the end of the study period.

### Implementation strategies

The success of the iCARE treatment intervention on improving testing and linkage to care for YMSM occurred despite a number of implementation barriers. Program staff described social and provider stigma, legal environment, safety concerns, unwelcoming environments of many HIV care sites, COVID-19 pandemic, and lack of adequate supplies of condoms and lubricants. In addition, the social media component had to address barriers of internet-based social networking, internet cost and overcome distrust by YMSM as well as the need for confidentiality and establishing trust. Many of these contextual barriers were addressed, or at least mitigated, through the implementation strategies employed by the iCARE Nigeria study team (Table 2) and are discussed in detail below.

**Table 2:** Overall iCARE Nigeria testing implementation strategies mapped to barriers and outcomes to inform the Implementation Research Logic Model

Overall iCARE Nigeria testing	Examples of barriers addressed or facilitators leveraged	Implementation outcomes supported
Stakeholder engagement including community organizations, serving the targeted community, healthcare workers, clinic managers. This occurred during the formative adaptation phase, throughout the study and for input into planning for scale-up	**Barriers** Stigma (provider) Health system barriers Program isolation or estrangement from other work designed to engage the target population **Facilitators** Presence of organizations working with the targeted population	Acceptability, feasibility
Hiring criteria for peers (members and non-members of the YMSM community, and requiring experience working with MSM)	**Barriers** Stigma (community) Legal barriers Confidentiality needs YMSM fear and distrust of non-YMSM members **Facilitators** Presence of Youth clubs (facilitator)	Reach, acceptability, feasibility, adoption, maintenance
Peer navigator training	**Barriers** Safety and confidentiality needs Fear of disclosure **Facilitators** Knowledge of YMSM concerns and needs	Reach, feasibility, adoption, fidelity, acceptability
Ongoing supervision	**Barriers** Distraction by areas of focus outside the protocol and personal agenda Safety, and confidentiality needs Emerging challenges (ex. COVID)	Feasibility, adoption, fidelity, maintenance
Data audit and continual feedback of recruitment and testing numbers and results to the team and individual PNS to inform strategy adaptation and improve and data quality and implementation	**Barriers** Incomplete, missing, or flawed data Need to adapt strategies (ex. use of different social media apps)	Feasibility, adoption, fidelity
Team-based problem solving and support	**Barriers** Stigma Safety Staff burn out and risk of attrition	Feasibility, adoption, fidelity, maintenance
Social media outreach		
Selection of existing social media sites and apps with free access and use for different goals (initial engagement (Grindr, Facebook) or connection to online chat community (WhatsApp)	**Barriers** YMSM fear and distrust of non-community members Security needs Cost of internet access **Facilitator** Comfort in engaging on existing social media platforms including those which were MSM-specific	Reach, acceptability, maintenance
Use of both YMSM-specific and generic social media where participants were already engaging		
Use of both open and closed group social media		
Adapting online content to target population needs and online costs and capacity, (e.g., refraining from sharing images or videos that would consume scarce resources during the downloading or streaming process)	**Barriers** Lack of existing knowledge resources responsive to YMSM needs Limited participant financial resources for maintaining online activities	Reach, acceptability, maintenance
Use of flexible and informal communication methods responsive to needs of the YMSM by PNs	**Barriers** Lack of trust by YMSM in engaging with new individuals Different needs based on individual readiness to test	Reach, acceptability
Testing and linkage navigation		
Establishing a process to confirm client identity before meeting with PN	**Barrier** Legal challenge PN safety False claims of YMSM status by participants	Feasibility, reach, acceptability, adoption
Flexibility in testing site locations and timing of encounters	**Barriers** Stigma (community) Legal barriers YMSM distrust of providers PN safety Client unavailability for testing during regular hours Safety and feasibility of social settings for pretest/post-test counseling	Feasibility, reach, acceptability, adoption
Training PNs in safety and protocols		
Active peer navigation for linkage to care for clients who test positive for HIV	**Barriers** Stigma (community, HCWs) Health system barriers (structure, location) Client denial of diagnosis and/or need for care Low self-efficacy for serostatus disclosure	Reach, acceptability, adoption
Provision of needed supplies through the grant (test kits, personal protective equipment, cellular data on mobile devices)	**Barriers** COVID YMSM risk-reduction needs Cost and availability of Smart phones for PNs	Reach, feasibility, acceptability, adoption
Navigation of clinets testing positive for HIV to preferred care site perceived as YMSM-friendly	**Barriers** Perceived high barrier for linkage to HIV care by YMSMs Stigma (community, HCWs) **Facilitator** Presence of YMSM-friendly sites	Acceptability, feasibility, adoption
Provision of identification (ID) cards to the PN	**Barriers** Legal barriers PN safety risks	Feasibility, adoption, maintenance
Documentation integrated into national testing system including reporting forms	**Barriers** Fear of disclosure	Acceptability, maintenance

*YMSM* young men who have sex with men, *PN* peer navigator, *HCW* health care worker

#### Hiring criteria and training of peer navigators

Recruitment of four peer navigators, including two from the key population and one cis-gender female, who all had high commitment to and experience working with the community, was identified as important for success. These program staff addressed barriers including legal environment, stigma, confidentiality needs and YMSM fear and distrust of non-YMSM members. Further, the peer navigators noted the importance that the outreach coordinator, who supervised the four peer navigators, was also from the community. While the female peer navigator had success in outreach for transgender women, she was not fully able to leverage the gender-sensitive Grindr and YMSM networks on Facebook limiting feasibility of some components of her outreach.

“*So having KP populations focal persons in this facility as primary workers goes a long way. …. enlightening the rest of the workers about the existence of these people and the possibility of them having to come around and they attending to them, and how they can actually attend to them to make them feel comfortable also goes a long way. So, having primary KP workers in this facility can make the facility KP-friendly*”. (peer navigator)

Initial training, ongoing supervision and retraining the peer navigators as a group were additional strategies that supported development of needed skills and created an environment for the peer navigators to share operational challenges and solutions, increasing the success of implementation outcomes. “*So I picked from that and we…in training…they chipped in a lot, and it has helped me along the way*” (Program staff). When reflecting on a difficult situation with a client, one peer navigator noted the value of the training he had received and ability to discuss with the team.

The use of team-based problem solving was an additional strategy, facilitated by the problem-solving mentality (of the peer navigators) about their job.

“*but one just needs to be ready, be prepared to answer questions, abusive question, question that demands immediate answer. ….To make sure…even if we…we…we are not expecting anything but be prepared and…be smart to give them a reasonable answer which can really convince them and actually believe you that oh, this thing is true. Because once you lose a client, you’ve ‘los[t]’ like ten clients*” (peer navigator).

The recruitment of peer navigators who had experience working with peers, supported by ongoing training from the program manager and lead investigator helped the peer navigators address barriers encountered in outreach and testing of the YMSM. The skill and commitment of the peer navigators was shown by the persistence and flexibility displayed for both the scheduling and location of testing as well as their understanding of what was needed to “transform from the virtual to the physical world”. They reported multiple examples where initial appointments were not kept, but sustained engagement ultimately resulted in acceptance of testing.

“*I have tested in so many places. We test at their homes, at times they can call you to come to their home, they can call you to a particular place or you make suggestion to them…for them that (is) ok*” (peer navigator).

These strategies helped ensure low turnover of staff, an important facilitator for success. Another important strategy for building community trust was the engaging and collaborating with key stakeholders including local comuninty-based organizations working with YMSM to help identify key population-friendly clinics which were welcoming and supportive of the peer navigators and to the clients they referred.

#### Social media

Respondents also identified the careful choice and use of different free publicly available social media apps and sites as a key strategy for the success in acceptability, adoption, feasibility, and effectiveness of the intervention. These included both MSM-specific apps (Grindr), more generic sites (Facebook but which included informal networks) and closed generic platforms for the more confidential (but still anonymous) groups (WhatsApp). The use of each of these social media tools tapped into their respective functionalities (e.g., geolocator function, closed groups, etc.) to achieve specific goals (outreach, linkage for testing, education, community building) in line with community preferences and established patterns of use by the YMSM. This differential use addressed many MSM-associated barriers including stigma, fear of disclosure, and safety. As one peer navigator noted, this selective use of different social media applications allowed the peer navigators to navigate clients from initial engagement to meeting for testing. “*I get most of my clients on Facebook and then I navigate them to WhatsApp for a more direct and proper conversation and follow up. So, it’s actually helpful*” .

Once trust was established on both the peer navigator and peer’s side, there was often a referral and connection to the online chat community (WhatsApp). The closed WhatsApp group was “*more interactive because it’s private*” and was used to continually educate the community as well as engage individual youth prior to testing. Project managers and peer navigators developed discussion topics related to the concerns and interests of the YMSMs. This strategy was important for reach and acceptability.

“*we know the kind of issues that are, that is peculiar to the community members. So, like some of them come down with STIs. So, we target those topics around STIs and anal warts and the like. And from there, we, we, like we enlighten them more, we create awareness and for those who need help, we encourage them to probably speak up and then send a private chat so we can take it up from there. So, those topics they came out of like… us knowing the needs of these people and we try to create topics around their needs.*”* (Program staff)*.

The confidentiality offered by WhatsApp was also identified as important for reach and acceptability and effectiveness to address distrust and fear of disclosure. The platform allowed for private chats between participants not comfortable disclosing issues such as sexually transmitted infections in a larger group chat. Those participants still benefitted from knowledge disseminated in the larger group format as well as information given for referral services. The topics discussed in closed groups were determined at a local level and implemented by staff; with a specific focus on health information and prevention science relevant to YMSM The group was also closely monitored with posted rules and regulations and the peer navigators given authority to remove difficult clients to maintain integrity of the group. Once in the group, youth could also voluntarily exit the group.

iCARE also established an informal process to confirm that individuals were members of the YMSM which contributed to acceptability and adoption and feasibility. As one peer navigator noted, “*I need to be sure that oh, this person, if he’s not a scam, if he’s not a Kito (*a term used by KPs to refer to people who exploit the LGBTQ population using blackmail or violence*), if he’s not so-so thing, all manner of things like that*”. One peer navigator described how the use of social media led to increased awareness of iCARE in the community which could support longer term sustainability. “*So, the group has gone far, it has really exposed iCARE—the name iCARE to the world that oh, iCARE is existing, and it’s a very nice group*”.

#### Testing strategies

A number of strategies were identified which contributed to the acceptability, adoption and reach of iCARE Nigeria testing intervention and overcoming barriers, particularly around stigma and the legal environment. These built on the hiring, training and supervision strategies. Using a public health approach to test all young men rather than screening for individual-level YMSM status reduced the risk of disclosure. Flexibility in testing site locations made the intervention more acceptable and increased feasibility and adoption by using places which were safe and preferred by the youth. Respondents identified the priority of confidential testing while letting the client choose preferred sites. They described the challenges related to performing the test in cramped spaces in ways required to maintain confidentiality. For example, not being able to spread out the test kit materials (e.g., on a park bench), which would be acceptable in a clinic setting. Locations where testing procedures were constrained included social settings like parties, as well as gas stations or in the client’s home.

“*You have to be smart; you have to be fit and don’t just carry everything and spread [testing materials] like you are in the office, or you are in the laboratory, no. There are…you can carry your test kit and everything, just spread it on where you are sitting and do it…do whatever you are doing, nobody will get to know that this is what you are doing……just…you are doing your test*” (peer navigator).

Importantly, the stakeholder engagement before the study started allowed the program to identify key population-friendly clinical sites for confirmatory testing and linkage to care as well as other community-based organizations serving YMSM. Establishment of close working relationships with these clinics combined with active navigation of HIV seropositive youth to them supported the high success rates of confirmatory testing and linkage to care. This strategy was critical to the acceptability and overall reach, acceptability, fidelity, and effectiveness. Peer navigators noted the risk of losing clients by referring to clinics which were not welcoming to YMSM or sensitive to their needs. The collaboration with these organizations also helped iCARE acceptance by the MSM community and groups working to support their needs.

Provision of needed supplies (test kits, personal protective equipment, cellular data on mobile devices and formal ID cards) was also an important strategy. Some of these supplies were needed to address challenges of the COVID-19 pandemic while others addressed challenges posed by stigma and legal environment. The identification (ID) cards provided by the study were critical to ensure the safety (and therefore feasibility and adoption) of the work due to the existing laws in Nigeria which prohibit same-sex marriage, and the public display of same-sex relationships. Peer navigators described examples of times when they were approached by police or other security men where the ID card provided proof of their official roles.

“*that’s why we have our ID cards, like whenever we are going. Of course, there was a day I was stopped by [the police]. So, going along with him, we were on the bike. Going there on bike, the policeman now stopped us. Why did he stop me? It was because of how the guy was dressed; …. The police now stopped us, he started interrogating. “Where are you guys going to?” I said, I’m going to work. Ah he now said ok, where do you work? Then I explained, I told where I work, and I told him the kind of work I do. ….So, after that, you…the next thing they will ask is, where is your ID card? Do you have your ID card?… I brought my ID card. With that, there’s no further question*” (peer navigator).

The start of the COVID-19 pandemic emerged as a new barrier, initially causing a break in iCARE Nigeria services. However the team recognized that the risk of HIV transmission did not stop (“*It doesn’t stop them having sex*” (peer navigator)), so began planning strategies to address this new barrier. Once COVID-related movement restrictions were lifted, the peer navigators were given PPE and training on infection control and prevention techniques. To further ensure ongoing adoption of testing, the peer navigators would bring additional masks for meeting with clients and learned how to do the testing while practicing appropriate social distancing.

Reflecting the challenges and fears of accessing care, the peer navigators also provided navigation for HIV negative clients for other care needs, and linkage to care for those who tested positive for HIV using sites identified as welcoming to YMSM. They also accompanied and advocated for their clients who needed additional care such as treatment for anal warts.

“*when they take a client for confirmatory (test) and then it comes out positive, they enroll, they make sure they still navigate the client to treatment and make sure they finish up with the treatment. After that, they still do follow-up for refills and other appointment*”*.* (Program Staff)

One supply gap identified was adequate supplies of condoms and lubricants due to costs and availability. Ensuring a supply of these products for the peer navigators to give to their clients was identified as an important strategy for incentivizing youth engagement while preventing acquisition of HIV and other sexually transmitted infections.

The combination of a number of these strategies, leveraging the characteristics of the peer navigators was core to the overall success in reach, acceptability, fidelity, adoption and contributed to the maintenance of the peer navigators with no turnover during the initial study period of 48 weeks.

An unexpected outcome of the implementation of the iCARE Nigeria testing project was a change in culture at the host institution. As a result of the hiring of and working with peer navigators from the key population, respondents reported that organization and clinic have become more welcoming to this group with clients testing positive willing to come for care. Talking about introducing a client to IDI through iCARE, one peer navigator noted.

*“iCARE office is for everybody.” …… I gave him the full confidence that you come…call me at any time if you want to come to my office. Then when he came, he saw everybody, he was like “wow!” I introduced them, “this is (program manager) he also works with iCARE, ….So, with that, you make him feel comfortable and relaxed that ok, I can really trust this people. Lo and behold, he has so many people that he refers to me, which I enroll them here in ID” *(peer navigator).

### Implementation research logic model

**Fig. 1:**
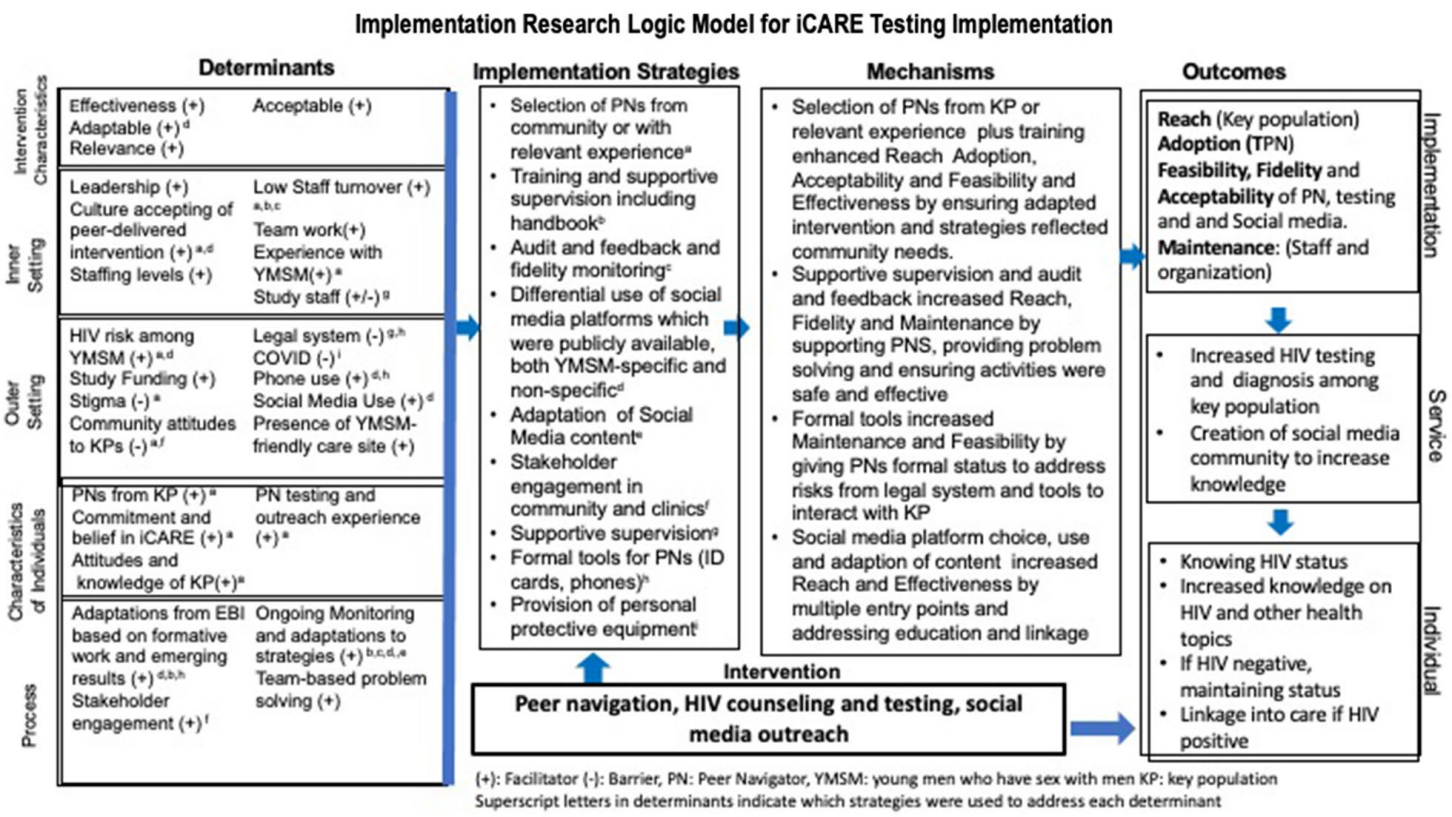
Implementation Research Logic Model for iCARE Nigeria Testing Intervention

The results were synthesized into an Implementation Research Logic Model to map from the barriers and facilitators through the strategies to the outcomes (Fig. 1). The use of this framework helped identify the mechanisms through which the strategies supported the implementation and effectiveness outcomes, which would help inform the next phase of the study. For example, Selection of peer navigators from targeted population or relevant experience plus training enhanced Reach, Adoption, Acceptability and Feasibility and Effectiveness by ensuring adapted intervention and strategies reflected community needs and through trust between peer navigators and clients.

## Discussion

Using mixed methods, we were able to expand understanding of the reported efficacy and implementation outcomes of a combined social media and peer navigator intervention in Ibadan to engage, test and link into care male youth focusing on YMSM. While there were multiple barriers at the community, health system and participant levels, we found that the strategies employed to implement the components of iCARE Nigeria testing intervention were important in achieving the implementation outcomes needed to achieve the overall program effectiveness. Use of the Implementation Research Logic Model, a tool increasingly used in implementation research, further allowed understanding of the linkage between the contextual factors (setting, population, etc.), strategies chosen, and the outcomes observed [8] .

Peer navigators have been widely studied in HIV treatment interventions and growing in use to enhance HIV prevention services, testing access, and linkage to treatment post testing in a number of countries and populations, although less frequently for YMSM in Africa [14–16]. Our findings are also similar to a peer navigator testing interventions for youth in Kenya, finding high acceptability (88% agreeing to testing) and effectiveness in identifying youth testing positive for HIV and linkage to care respectively [14]. While these interventions shared some strategies (hiring peers from the community, training, provision of condoms), others such as integration of social media were different, as well as some of the approaches needed to address legal and safety barriers encountered in our pilot setting.

Many of the strategies employed by the iCARE Nigeria study team are frequently used in implementing community-targeted programs including the differential use of social media, stakeholder engagement and choosing implementers from the targeted community [17–19]. For example, stakeholder engagement in both the formative and implementation phases was important as it is for vulnerable and highly stigmatized populations such as YMSM in Nigeria and elsewhere [17].

The strategies to identify, hire, train and support the peer navigators also promoted success. The quantitative results reflect high acceptability, adoption, and fidelity, with 100% retention of the peer navigators through considerable challenges including COVID-19. The success of these strategies was reflected in the peer navigators’ skills in navigating the YMSM along the path from virtual social media platforms to testing, and linkage to care (for those testing positive) as well as their commitment to serving their communities. In addition, retention was high compared with a study of multiple sites implementing outreach for YMSM in the US with reported 57% retention [20]. Results from that study also found challenges for outreach workers functioning as peer navigators for YMSM in the United States and the need for protocols and ongoing supervision, strategies identified as important for success of the iCARE Nigeria testing intervention.

Publicly available social media apps, including YMSM-specific and non-population-specific ones that allow private secure groups have also been used successfully to reach MSM by other research groups [21]. However other studies using social media have found variable results depending on the level of engagement [19]. The goal of empowering YMSMs through awareness generation was facilitated by choosing topics relevant to the youth as determined by peer navigators and the study team members, plus requests of the youth themselves. Overall, program initiatives supported the acceptability of delivering education through the WhatsApp groups in particular.

The strategies to ensure safety of the peer navigators and the community served were important for successful implementation and effectiveness of the testing intervention. These included careful training and supervision of the peer navigators and problem-solving at the individual level to decide where and how testing was initiated and completed, along with collaboration with community-based organizations working with YMSMs. In addition, ID cards for the peer navigators to identify them as members of the iCARE and designed processes to confirm that newly contacted individuals were part of the key population. This last strategy was essential, given the risk of individuals falsely self-identifying as YMSM in order to identify and report legitimate YMSM to law enforcement authorities because same sex relationships are still criminalized in Nigeria [22, 23].

## Limitations

The iCARE study design led to a number of limitations in this evaluation of the implementation strategies and context. Notably, because of the anonymity of the recruited young men, which was important for safety, and COVID-19 precautions, we were not able to interview any of the recruited or tested young men and interviewed only a limited number of peer navigators and study staff. However, despite the small number of interviews, thematic saturation was achieved and provided insight to the quantitative outcomes. As with all qualitative work, the reported activities were not specifically verified although triangulation with the quantitative data were confirmatory. In addition, for safety and anonymity, we only collected acceptability of testing among individuals who agreed to meet the PNs, which likely over-estimated acceptability across the entire population interacting with the iCARE testing intervention.

In conclusion iCARE Nigeria’s pilot combination HIV testing intervention’s high acceptability, fidelity and adoption were linked to implementation of strategies which addressed many challenging contextual factors, including social stigma, legal barriers surrounding MSM behavior, and the COVID-19 pandemic. The intervention is now being tested in three additional Nigerian cities building on the lessons from the pilot phase. The use of implementation research methods including the Implementation Research Logic Model are important to create generalizable knowledge from pilots to inform scale-up of behavioral and health systems interventions needed to end the HIV epidemic in Nigeria and in other countries facing similar challenge.

### Supplementary Information

**Additional file 1.** Research checklist: COREQ (COnsolidated criteria for REporting Qualitative research) checklist

**Additional file 2.** Overall iCARE testing intervention strategies mapped to barriers and outcomes. Table detailing the strategies mapped to the barrier addressed and outcomes achieved.

## Data Availability

Because of the sensitivity and inability to fully protect identify, qualitative data are not available. Deidentified participant quantitative data are available on request from lkuhns@luriechildrens.org.
