# Supplementary material for: Mixed methods implementation research to understand success of intensive combination approach to roll back the epidemic in Nigerian adolescents) (iCARE Nigeria) HIV testing uptake and linkage to care among young men focusing on young men who have sex with men in Ibadan
Source: AIDS Res Ther. 2023 Oct 30;20:75. doi: 10.1186/s12981-023-00574-4 (PMC10617228; doi:10.1186/s12981-023-00574-4)
Supplement: Supplementary file 1 — Additional file 1. Research checklist: COREQ (COnsolidated criteria for REporting Qualitative research) checklist [file 12981_2023_574_MOESM1_ESM.docx]

**Additional file 1**

| **Additional File 1: Definitions of implementation outcomes** | |
| --- | --- |
| Outcomes | Definition |
| Feasibility: | Ability to implement the program components   - Ability to hire and retain peer navigators - Ability to provide testing in the community - Online chat group designed and implemented and continued weekly on generic social media platforms weekly, led by peer navigators and program leads. |
| Fidelity: | Adherence to established testing protocol   - Provision of pre-test counseling before undergoing rapid testing, followed by post-test counseling based on test results - Referral for those testing positive for HIV referred for care - Linkage to care for initiation of antiretroviral treatment within 30 days of confirmation of positive HIV test |
| Adoption: | Delivery of testing by peer navigators   - Peer navigators conducting in-person outreach, testing, and navigation - Peer navigators participating in social media-based activities, both key population specific and generic social media platforms |
| Acceptability: | Agreement to testing when offered in person by peer navigators and engagement in social media   - Individuals offered testing by peer navigator who accept testing - Number engaged in social media-based engagements |
| Reach | Targeted population reached for testing   - Population engaging in testing reflects the priority group (young men focusing on young men who have sex with men) |
| Maintenance | Ability of the peer navigators to continue to provide the testing outreach and delivery throughout the study period |
